# Expression of tissue factor in non-small-cell lung cancers and its relationship to metastasis

**DOI:** 10.1038/sj.bjc.6690073

**Published:** 1999-02

**Authors:** M Sawada, S Miyake, S Ohdama, O Matsubara, S Masuda, K Yakumaru, Y Yoshizawa

**Affiliations:** Department of Pulmonary Medicine, Tokyo Medical and Dental University, 1-5-45, Yushima, Bunkyo-ku, Tokyo, 113-8519, Japan; First Department of Internal Medicine, Tokyo Medical and Dental University, 1-5-45, Yushima, Bunkyo-ku, Tokyo, 113-8519, Japan; Department of Pathology, National Defense Medical College, 3-2, Namiki, Tokorozawa, Saitama, 359-0042, Japan; Department of Surgery, Tokyo Teishin Hospital, 2-14-23, Fujimi, Chiyoda-ku, Tokyo, 102-0071, Japan; Department of Pathology, Tokyo Teishin Hospital, 2-14-23, Fujimi, Chiyoda-ku, Tokyo, 102-0071, Japan

**Keywords:** tissue factor, lung cancer, non-small-cell lung cancer, metastasis

## Abstract

Tissue factor (TF) is an initiator of the extrinsic cascade of blood coagulation. Although recent studies have revealed a relationship between metastatic properties and TF expression in some neoplastic cells, the significance of TF in lung cancer, especially in non-small-cell lung cancer (NSCLC), is still unclear. In this study, TF was detected in NSCLC cell lines by functional study, Western blot analysis and immunocytochemical staining. TF levels in eight NSCLC cell lines were also quantitated by enzyme-linked immunosorbent assay (ELISA), and TF expression was evaluated in 55 specimens of surgically resected NSCLCs. NSCLC cell lines derived from metastatic lesions produced high levels of TF (48.3 ± 23.5 ng 10^−6^ cells, mean ± s.e.m.), whereas those derived from primary lesions produced low levels of TF (0.2 ± 0.1 ng 10^−6^ cells). Immunohistochemical studies disclosed significantly stronger staining for TF in cells from NSCLC patients with metastasis than in those without metastasis. Among the 28 patients with metastasis, ten were strongly positive, 16 were moderately positive and two were negative for TF. In contrast, among the 27 patients without metastasis, only two were strongly positive, 18 were moderately positive and seven were negative for TF. Therefore, malignant cells from patients with lung cancer produce various levels of TF, and TF may play an important role in the metastatic process. © 1999 Cancer Research Campaign


*British Journal of Cancer* (1999) **79**(3/4), 472–477
© 1999 Cancer Research Campaign
Article no. bjoc.1998.0073

### Expression of tissue factor in non-small-cell lung cancers and its relationship to metastasis

**M Sawada[1,2], S Miyake[1,2], S Ohdama[1,2], O Matsubara[3], S Masuda[4], K Yakumaru[5] and Y Yoshizawa[1,2]**

[1]Department of Pulmonary Medicine and [2]First Department of Internal Medicine, Tokyo Medical and Dental University, 1-5-45, Yushima, Bunkyo-ku, Tokyo 113-8519, Japan; [3]Department of Pathology, National Defense Medical College, 3-2, Namiki, Tokorozawa, Saitama 359-0042, Japan; [4]Department of Surgery and [5]Department of Pathology, Tokyo Teishin Hospital, 2-14-23, Fujimi, Chiyoda-ku, Tokyo 102-0071, Japan

**Summary** Tissue factor (TF) is an initiator of the extrinsic cascade of blood coagulation. Although recent studies have revealed a relationship between metastatic properties and TF expression in some neoplastic cells, the significance of TF in lung cancer, especially in non-small-cell lung cancer (NSCLC), is still unclear. In this study, TF was detected in NSCLC cell lines by functional study, Western blot analysis and immunocytochemical staining. TF levels in eight NSCLC cell lines were also quantitated by enzyme-linked immunosorbent assay (ELISA), and TF expression was evaluated in 55 specimens of surgically resected NSCLCs. NSCLC cell lines derived from metastatic lesions produced high levels of TF (48.3 ± 23.5 ng $10^{-6}$ cells, mean ± s.e.m.), whereas those derived from primary lesions produced low levels of TF (0.2 ± 0.1 ng $10^{-6}$ cells). Immunohistochemical studies disclosed significantly stronger staining for TF in cells from NSCLC patients with metastasis than in those without metastasis. Among the 28 patients with metastasis, ten were strongly positive, 16 were moderately positive and two were negative for TF. In contrast, among the 27 patients without metastasis, only two were strongly positive, 18 were moderately positive and seven were negative for TF. Therefore, malignant cells from patients with lung cancer produce various levels of TF, and TF may play an important role in the metastatic process.

**Keywords:** tissue factor; lung cancer; non-small-cell lung cancer; metastasis

In Japan, lung cancer is the leading cause of cancer death in men and the second leading cause, after stomach carcinoma, in women. Lung cancers are classified into several groups, including small-cell lung cancer (SCLC) and non-small-cell lung cancer (NSCLC). Although the most successful treatment for the latter group is surgical removal of the tumour, the average 5-year survival rate after complete resection is approximately 60–85%, 45% and 30% for stage I, II and IIIA disease respectively (Mountain, 1993). In addition, there is a marked variation in outcome among different individuals. Thus, it is desirable to obtain additional information on the phenotype and pathology of lung cancer to aid characterization of these tumours.

Tissue factor (TF) is a normal component of the haemostatic system. Initiation of blood coagulation is efficiently mediated by TF, the cell-surface receptor for the coagulation zymogen factor VII (FVII) and the receptor and co-factor for the serine protease factor VIIa (FVIIa). Protein–protein interactions between FVIIa and TF are considered essential for the co-factor functions, which involve the catalytic enhancement of FVIIa-mediated proteolytic activation of the zymogen substrates factors X and IX (Nemerson, 1988). TF is normally expressed in the adventitial layer and mesenchymal-appearing cells of large blood vessels (Drake et al, 1989; Wilcox et al, 1989), providing a barrier against blood loss into the extravascular space.

*Received 1 September 1997*
*Revised 8 April 1998*
*Accepted 16 April 1998*

*Correspondence to*: Y Yoshizawa, Department of Pulmonary Medicine, Tokyo Medical and Dental University, 1-5-45, Yushima, Bunkyo-ku, Tokyo 113-8519, Japan

Although TF has also been found in several types of neoplastic cells (Callander et al, 1992), and there appears to be a relationship between TF expression and the metastatic properties of tumours (Mueller et al, 1992), we have little information on TF expression in lung cancer, especially in NSCLC. In this study, we first examined NSCLC cells in culture and assessed the expression of TF by means of functional study, Western blot analysis and immunocytochemical staining. We then used a sensitive enzyme-linked immunosorbent assay (ELISA) to quantitate TF antigen (Ag) in a wide variety of lung cancer cell lines and examined whether the levels of TF are associated with the characteristics of the original tumour. We also immunohistochemically investigated the expression of TF in fresh-frozen specimens of lung cancers.

### MATERIALS AND METHODS

### Lung cancer cell lines and cell characteristics

Eight cell lines of human NSCLC were provided by the Health Science Research Resources Bank: five cell lines derived from adenocarcinoma (PC-3, ABC-1, RERF-LC-OK, RERF-LC-MS, and A549) and three from squamous cell carcinoma (EBC-1, LC-1sq, and LK-2). The characteristics of the cancer cells are shown in Table 1 (Smith et al, 1977; Hiraki et al, 1982; Itoh et al, 1991; Kishimoto et al, 1992). The cancer cells were maintained in Dulbecco's modification of Eagle's minimum essential medium or in RPMI-1640 with 10% heat-inactivated fetal bovine serum (FBS).

### TF and anti-TF antibodies

Recombinant human TF and mouse monoclonal antibodies (mAbs) (TF8-5G9 and TF9-6B4) specific for human TF were

**Table 1** Levels of tissue factor in cell lines derived from human non-small-cell lung cancer

| | Characteristics of original tumour | | | Tissue factor (ng $10^{-6}$ cells) | |
|---|---|---|---|---|---|
| | **Histological type** | **Clinical stage** | **Source** | **Cell lysate** | **Culture medium** |
| Adenocarcinoma | | | | | |
| PC-3 | P/D Ad | IV | Lymph node | 130.68 ± 11.06 | 6.70 ± 0.65 |
| ABC-1 | W/D Ad | IV | Pleural effusion | 70.55 ± 5.00 | 8.69 ± 1.34 |
| RERF-LC-OK | Ad | | Pleural effusion | 19.46 ± 0.94 | 0.82 ± 0.16 |
| RERF-LC-MS | Ad | | Pleural effusion | 16.20 ± 1.06 | 1.44 ± 0.10 |
| A549 | W/D Ad | I | Primary lesion | 0.10 ± 0.02 | 0.01 ± 0.01 |
| Squamous cell carcinoma | | | | | |
| EBC-1 | P/D Sq | IV | Skin metastasis | 4.52 ± 0.38 | 0.82 ± 0.05 |
| LC1sq | P/D Sq | I | Primary lesion | 0.42 ± 0.04 | 0.12 ± 0.03 |
| LK2 | P/D Sq | I | Primary lesion | 0.17 ± 0.01 | 0.04 ± 0.01 |

P/D, poorly differentiated; W/D, well differentiated; Ad, adenocarcinoma; Sq, squamous cell carcinoma; TF Ag in the cell lysates and soluble TF Ag in the culture media were measured by ELISA. TF Ag level is expressed as mean ± s.d. See Materials and methods for details.

obtained from Corvas International (San Diego, CA, USA). Human placental TF was obtained from Behringwerke (Thromborel S, Marburg, Germany). Mouse mAbs (ADI 4504 and 4509) and rabbit polyclonal antibody (pAb) (ADI 4502) specific for human TF were obtained from American Diagnostica (Greenwich, CT, USA). The specificity of these mAbs has been described in detail elsewhere (Morrissey et al, 1988; Ruf and Edgington, 1991; Ruf et al, 1991). Antigenic TF expression detected immunohistochemically with the use of these TF-specific mAbs (ADI 4504 and 4509) was reported to be correlated with functional TF expression (Contrino et al, 1994).

### Analysis of procoagulant activity in clotting assay

Confluent lung cancer cells in 100-mm culture dishes were suspended in phosphate-buffered saline (PBS, pH 7.4) and the suspensions were adjusted to $1 \times 10^6$ cells $ml^{-1}$ in PBS. Cell suspensions to be evaluated for total cellular procoagulant activity were frozen and thawed three times and sonicated. Cell suspension (0.1 ml, $1 \times 10^5$ cells) was added to 0.1 ml of pooled human normal plasma. After incubation at 37°C for 2 min, 0.1 ml of 25 mmol$l^{-1}$. $CaCl_2$ was added and plasma recalcification time was determined with a semiautomatic coagulometer (CA-100, Sysmex, Kobe, Japan). TF co-factor activity was quantitated by reference to standard curves constructed with placental TF. TF activity yielding a 50-s recalcification time was defined as 1 unit per ml. The values given are the means ± s.d. for quadruplicate samples.

### Neutralization test

Each lung cancer cell suspension (0.5 ml) was mixed and incubated with an equivalent volume of anti-TF mAb, ADI 4509, at a concentration of 50 μg $ml^{-1}$ at 37°C for 1 h. The clotting time of each mixture was measured by the recalcification method as described above. Control assays were performed with the use of preimmune mouse IgG.

### Immunoblotting analysis

Confluent lung cancer cells in 100-mm culture dishes were rinsed with PBS three times and disrupted by incubation with 0.5% Triton X-100 in PBS for 60 min. Clear lysates were then obtained by centrifugation for 5 min at 7000*g*. These samples were subjected to 12.5% sodium dodecyl sulphate polyacrylamide gel electrophoresis (SDS-PAGE) under reducing conditions. As protein content may vary greatly among cell lines, we adjusted the cell number, instead of the protein content, for SDS-PAGE. The lysates from $1.0 \times 10^5$ cells were applied on each lane. Purified recombinant TF and placental TF were used as positive controls. After separation by SDS-PAGE, these samples were electrophoretically transferred to polyvinylidene difluoride membranes (Immobilon-P, Millipore Corp., Bedford, MA, USA). The transferred membranes were then blocked by incubation in PBS containing 5% low-fat milk for 1 h, washed in the washing buffer and incubated with the appropriate mAb (TF8-5G9, ADI 4509 and a mixture of all mAbs described above) overnight. After washing, the membranes were incubated with peroxidase-conjugated anti-mouse IgG (Bio-Rad Laboratories, Hercules, CA, USA) for 1 h, followed by the addition of 3-3′-diaminobenzidine hydrochloride as substrate.

**Table 2** Tissue factor expression in surgically resected non-small-cell lung cancers

| Characteristics | Number of positive/total number tested |
|---|---|
| Total | 46/55 |
| Male | 31/36 |
| Female | 15/19 |
| Histological type | |
| Adenocarcinoma | 32/38 |
| Well differentiated | 10/12 |
| Moderately differentiated | 13/16 |
| Poorly differentiated | 9/10 |
| Squamous cell carcinoma | 12/15 |
| Well differentiated | 3/3 |
| Moderately differentiated | 2/2 |
| Poorly differentiated | 7/10 |
| Large-cell carcinoma | 2/2 |
| Pathological stage | |
| Stage I | 17/24 |
| Stage II | 5/5 |
| Stage IIIA | 17/17 |
| Stage IIIB | 5/5 |
| Stage IV | 2/4 |

*© Cancer Research Campaign 1999*

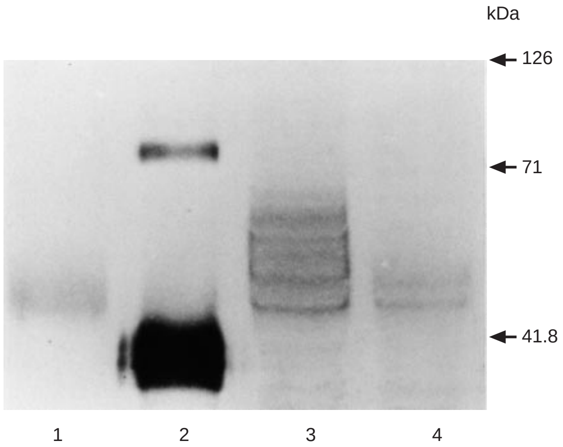

**Figure 1** Western blot analysis of lung cancer cells with anti-TF Ab. Detergent-soluble protein samples of lung cancer cells were analysed on a 12.5% SDS-polyacrylamide gel using a mixture of mAbs against TF. The molecular size markers used were β-galactosidase (126 kDa), bovine serum albumin (71 kDa) and carbonic anhydrase (41.8 kDa). Positions of the markers are indicated by arrows. The mixture of anti-TF mAbs identified the reduced form of recombinant TF as multiple bands at approximately 36–46 kDa (lane 2) and placental TF at 48 kDa (lane 1). TF detected in PC-3 cells producing high levels of TF on ELISA exhibited several intense bands distributed from about 46–64 kDa (lane 3), whereas TF in ABC-1 was determined as a doublet at approximately 46 kDa and 49 kDa (lane 4)

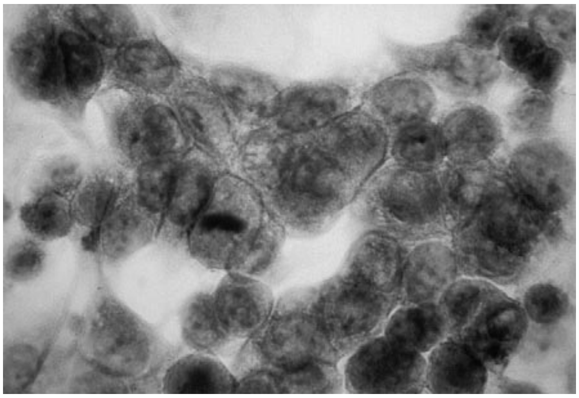

**Figure 2** Immunocytochemical staining for TF in cultured human lung cancer cell lines. Positive staining is indicated by the dark-brown reaction products. The presence of TF along the cell membrane is clearly demonstrated in adenocarcinoma cell line, ABC-1 (× 400)

### Immunocytochemical staining of TF from cultured human lung cancer cell lines

Lung cancer cells were cultured in culture plates (LAB-TEK, Miles Scientific, Elkhart, IN, USA), and the plates were washed with PBS three times. After the cells were fixed in methanol, the plates were incubated with 3% hydrogen peroxide in methanol for 5 min to block endogenous peroxidase activity. The plates were treated with 5% bovine serum albumin (BSA) and incubated with the appropriate mAb, TF8-5G9, overnight at 4°C. They were then washed with PBS, and mAb binding was visualized with the labelled streptavidin–biotin method (Dako, Carpinteria, CA, USA). The specificity of immunostaining was confirmed by negative control, using non-immune mouse IgG in place of the primary mAb and by preabsorption of each mAb with the placental TF (50 × molar excess).

### Measurement of TF antigen in cell lysates and culture media

Lung cancer cells were plated at a density of 0.7–1.0 × $10^5$ cells per well and grown for 4 days in 24-well culture plates at 37°C in a humidified atmosphere of 5% carbon dioxide in air. The culture medium was centrifuged at 1500 r.p.m. for 10 min and the supernatant was used for the assay of TF levels in the culture medium. Cell pellets in the plate were washed with PBS three times and disrupted by incubation with 400 μl of 0.5% Triton X-100 in PBS for 60 min. Then, the plates were centrifuged at 2500 r.p.m. for 20 min, and the supernatant of the cell lysates was analysed for TF levels.

Total TF Ag in the cell lysates and soluble TF Ag in the culture media were measured by ELISA using two anti-TF mAb, TF8-5G9 and TF9-6B4, as previously described (Koyama et al, 1994). TF concentration was determined in four samples of each cell line. The cell number was counted in four samples of each cell line and TF Ag level was expressed in terms of ng per $10^6$ cells.

### Immunohistochemical studies of clinical specimens

A total of 55 patients with lung cancer who underwent lung resection at Tokyo Medical and Dental University or Teishin Hospital from August 1992 to January 1997 were studied (Table 2). Histological diagnoses were made on the basis of sections stained with haematoxylin and eosin. The specimens included 38 adenocarcinomas, 15 squamous cell carcinomas and two large-cell carcinomas. The pathological staging of each tumour was determined according to the TNM system (Hermanek and Sobin, 1992). The classification of staging in the patients was as follows: stage I, 24 patients; stage II, 5 patients; stage IIIA, 17 patients; stage IIIB, 5 patients; stage IV, 4 patients. Three of the 17 stage IIIA patients were T3N0M0. Therefore, of the 55 patients, 27 had neither hematogenous nor lymphogenous metastasis. None of the patients had received chemotherapy before surgery. Informed consent for lung resection was obtained from all patients. Immediately after lung resection, tissue samples were embedded in OCT compound (Tissue-Tek, Miles Scientific, Elkhart, IN, USA) and frozen in liquid nitrogen for cryostat sectioning. For immunohistochemical examination, 6-μm-thick cryostat sections were fixed in cold acetone for 5 min or in 2% paraformaldehyde for 1 h and staining was performed in a similar manner as described for immunocytochemical staining with TF-specific mAb (TF8-5G9, ADI 4504 and 4509) and pAb (ADI 4502).

All specimens were read blind by two experienced pathologists. The specimens were compared with negative controls, and the percentage of positively stained neoplastic cells was estimated. The results were graded as follows: negative (–), completely negative; moderately positive (+), the percentage of positive neoplastic cells was under 90%; and strongly positive (++), the percentage of positive neoplastic cells was 90% or higher.

### Statistical analysis

The data were analysed with the chi-square test, and *P* values less than 0.05 were considered to indicate statistical significance.

 *© Cancer Research Campaign 1999*

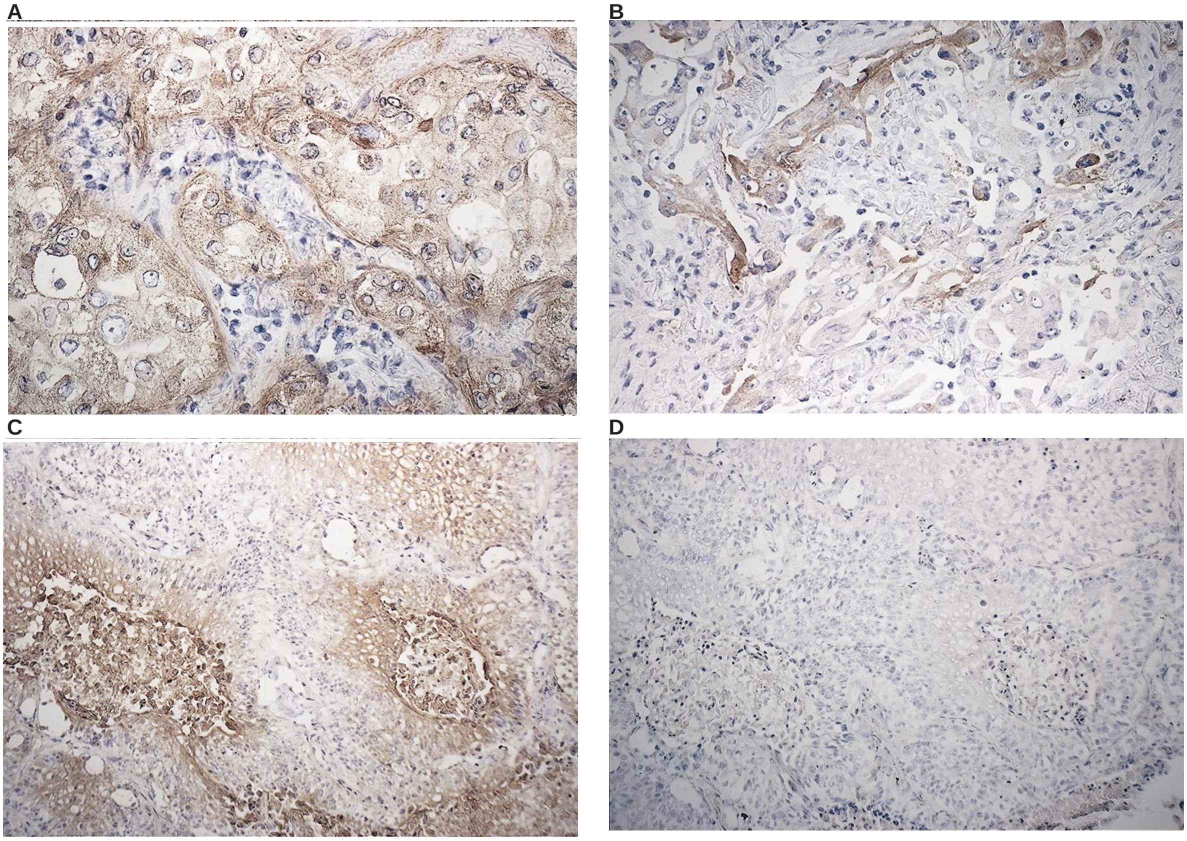

**Figure 3** Immunohistochemical staining for TF in surgically resected lung cancer tissues. (**A**) Large cell carcinoma. All tumour cells stain brightly, and this case is graded as strongly positive (++) (× 84). (**B**) Poorly differentiated adenocarcinoma. In this case, a heterogeneous pattern of staining is observed. This case is graded as moderately positive (+) (× 72). (**C**) Moderately differentiated squamous cell carcinoma. This case is graded as moderately positive (+). TF-positive staining among the squamous cell carcinoma cells is apparent in mature central keratinized areas (× 36). (**D**) Specificity control, the same case as Figure 3 (**C**). The specificity of the staining was verified by blocking this reaction by pre-absorption of the probe with purified placental TF (× 36)

### RESULTS

### Procoagulant activity of lung cancer cells and neutralization test

Procoagulant activities of three adenocarcinoma cell lines were determined by the recalcification time. Procoagulant activities were 45.1 ± 0.8 U $ml^{-1}$, 0.6 ± 0.07 U $ml^{-1}$, and 0.06 ± 0.01 U $ml^{-1}$ for PC-3, ABC-1, and A549 respectively. These procoagulant activities were neutralized by preincubation of cells with an anti-TF mAb (99.5% inhibition for PC-3 and 99.1% for ABC-1), showing that TF contributes to the cell procoagulant status.

### Western blot analysis of lung cancer cell lysates with anti-TF Ab

The presence of TF in lung cancer cells (PC-3 and ABC-1 from adenocarcinoma) was determined by immunoblotting with the mAb specific for human TF (Figure 1). The mAbs (ADI 4509 and TF8-5G9) and the mixture of all mAbs described above identified the reduced form of recombinant TF as multiple bands at approximately 36 kDa to 46 kDa and a minor 75-kDa band, which may represent a dimeric form, and placental TF at 48 kDa. TF in ABC-1 appeared as a doublet at about 46 kDa and 49 kDa, which is similar to the size of placental TF, whereas TF detected in PC-3 cells producing high levels of TF on ELISA exhibited several intense bands distributed from about 46 to 64 kDa.

The recombinant TF used in this study was synthesized by Chinese hamster ovary (CHO) cell lines. Although recombinant human TF expressed in *Escherichia coli* lacks post-translational disulphide bond formation and glycosylation, the CHO cell lines express glycosylated TF, which is reported to show multiple bands with reduced samples. Size heterogeneity of recombinant TF is due to differences in glycosylation of a single protein. The role of post-translational glycosylation in size heterogeneity has been confirmed by showing that reduced heterogeneity results in a single 36-kDa band of TF, when biosynthesis is done in the presence of tunicamycin to inhibit N-linked glycosylation (Rehemtulla et al. 1991).

TF in lung cancer cells also exhibited several bands. This may have been caused by the presence of TF in a variety of forms with different qualities and degrees of glycosylation and by the presence of TF as a dimeric form on the surface of cells (Fair and MacDonald, 1987).

### Immunocytochemical evaluation of TF

Two adenocarcinoma cell lines (ABC-1 and A549) and one squamous cell carcinoma cell line (EBC-1) were used for immunocytochemical studies of TF. Anti-TF mAb reacted strongly with ABC-1

*© Cancer Research Campaign 1999*

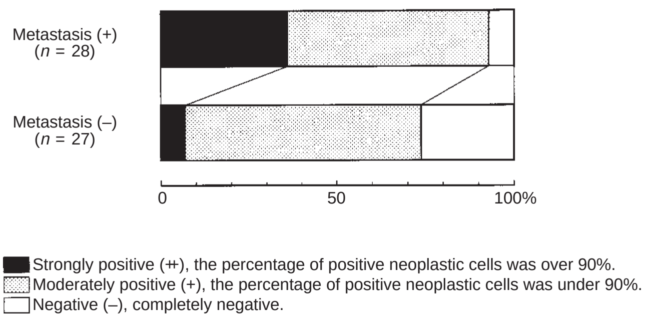

**Figure 4** Comparison of TF expression between groups with and without metastasis. TF expression is significantly different between the groups ($P < 0.05$)

and EBC-1 but weakly with A549. ABC-1 cells were homogeneously stained along the cell membrane (Figure 2), whereas EBC-1 cells were heterogeneously stained: some cells were stained strongly and other cells were stained very weakly.

### TF Ag in cell lysates and culture media of lung cancer cell lines

The levels of TF Ag in cell lysates and culture media of eight cell lines of human NSCLC were examined (Table 1). TF Ag was detected in both cell lysates and culture media of all lung cancer cell lines assayed. Most adenocarcinoma cell lines, except for A549, produced high levels of TF. Five of eight NSCLC cell lines (PC-3, ABC-1, RERF- LC-OK, RERF-LC-MS and EBC-1) were derived from metastatic lesions or malignant cells in pleural effusion. These cell lines produced high levels of TF ($48.3 \pm 23.5$ ng $10^{-6}$ cells mean ± s.e.m.; range 4.52–130.68), whereas the cell lines derived from, primary lesions (A549, LC1sq and LK2) produced low levels of TF ($0.2 \pm 0.1$ ng $10^{-6}$ cells; range 0.10–0.42).

### Immunohistochemical studies of TF in clinical specimens

In all cases examined, normal bronchial epithelial cells were positive for TF, staining more strongly in basally located cells, and the adjacent submucosa was negative. Endothelia of capillaries and of small to medium-sized blood vessels did not react at any site. Alveolar epithelial cells, especially type II alveolar pneumocytes, showed positive staining for TF. These results are consistent with previous observations (Drake et al, 1989).

Lung cancer tissues from 55 patients were examined for expression of TF (Figure 3). All mAbs and pAb reacted with each of the lung cancer specimens in a similar manner. The specificity of the staining was verified by blocking this reaction by pre-absorption of the probe with purified placental TF (Figure 3C and D). A heterogenous and studded pattern of staining was sometimes observed within the positive sections. Therefore, the specimens were evaluated semiquantitatively by calculating the percentage of positively stained neoplastic cells. The result of staining was scored from (–) to (++) (see Materials and methods for details).

The relationship between TF expression and pathological characteristics was analysed in each sample (Table 2). Of the 55 patients examined, 46 (84%) were positive for TF; 32 of 38 (84%) adenocarcinomas were positive for TF, 12 of 15 (80%) squamous cell carcinomas were positive and two of two (100%) large-cell carcinomas showed positive staining for TF. Strongly positive staining (++) of neoplastic cells was observed in 11 of 38 (29%) adenocarcinomas as compared with only one of 17 (6%) other histological types of tumours.

There was a significant difference in TF expression between the group that was positive for haematogenous or lymphogenous metastasis (28 patients) and the group that was negative for metastasis (27 patients). Of the 28 patients who had metastasis, 26 (93%) showed positive TF expression; of the 26, 10 were strongly positive (++) and 16 were moderately positive (+). Of the 27 patients who had no metastasis, 20 (74%) showed positive TF expression; of the 20, only two were strongly positive (++) and 18 were moderately positive (+) ($P < 0.05$) (Figure 4).

### DISCUSSION

Although TF has been found in several types of neoplastic cells (Silberberg et al, 1989; Callander et al, 1992), TF expression in lung cancer, especially in NSCLC, remains controversial. Immunohistochemically, Callander et al (1992) examined fresh-frozen specimens and showed that lung cancers, including NSCLC, expressed TF. In contrast, Ornstein et al (1991) examined acetone-methylbenzoate-xylene (AMeX)-fixed specimens and reported that TF expression was rare among NSCLCS. Contrino et al (1996) reported later that the previous failure to demonstrate TF expression immunohistochemically could have been due to a technical issue related to choice of tissue fixation techniques. Therefore, we investigated TF expression in fresh-frozen specimens to avoid this technical problem; we detected TF expression in 46 of 55 specimens of NSCLC. Furthermore, functional study (clotting time), Western blot analysis and immunocytochemical study also indicated that TF is present in NSCLC cells in culture. We therefore conclude that NSCLC cells produce various levels of TF.

Recently, several investigators have suggested a causal relationship between TF expression and the metastatic properties of tumours. Adamson et al (1994 *a,b*) demonstrated that procoagulant activity characterized as a TF–FVIIa complex reflects the malignant phenotype in both human cases and an animal model of prostate cancer. Mueller et al (1992) reported that metastatic melanoma cells expressed higher levels of TF than did non-metastatic cells and that inhibition of TF receptors resulted in a significant reduction in melanoma cells retained in the lung in a murine model. Although there are many reports on the role of TF in tumour growth, most previous studies have included only a few types of cell lines and studies of clinical specimens are rare. Therefore, we measured TF in a wide variety of NSCLC cell lines and found that human NSCLC cell lines derived from metastatic or disseminated lesions produced high levels of TF in vitro. Difficulties in comparing the biological properties of cell lines with those of their original tumours and in comparing the properties of different cell lines with each other have often been discussed. It has also been suggested that the properties of cell lines might change with passage number and culture conditions. For example, TF expression is rapidly induced in various types of cells by serum containing platelet-derived growth factor, fibroblast growth factor, transforming growth factor β and epidermal growth factor (Mackman, 1995). Therefore, we additionally studied surgically resected specimens to further investigate the role of TF in tumour growth and metastasis. We found a significantly stronger staining for TF in human NSCLC specimens with metastasis than

© Cancer Research Campaign 1999

in those without metastasis, suggesting that TF is associated with the spread of NSCLC.

The exact mechanisms by which TF is involved in metastasis are not clear. Bromberg et al (1995) reported that metastasis occurred in mice receiving injections of coagulation-defective TF mutant melanoma cells, suggesting that TF promotes metastasis by a pathway independent of blood coagulation. Zhang et al (1994) demonstrated that transfecting an antisense DNA sequence for the TF gene into sarcoma cells resulted in a marked reduction in their ability to secrete vascular endothelial growth factor (VEGF); this suggests that TF expression in tumours may have a direct or indirect effect, or both, on neovascularization. On the other hand, the effect of TF on metastasis is known to be mediated in part by products of the coagulation cascade. Fibrin generated around tumours might protect malignant cells from host defence mechanisms (Gunji and Gorelik, 1988) and thrombin might support the invasion of tumour cells by degrading the subendothelial matrix (Esumi et al, 1991). Procoagulant activity might facilitate cell adherence to the endothelium, extravasation of tumour cells, or proliferation of tumour cells at the site of a secondary deposit (Gasic, 1984).

Cultured lung cancer cells examined in the present study showed various levels of procoagulant activities. Two different procoagulants, TF and cancer procoagulant, have been identified as possible initiators of the coagulation cascade in malignant disease (Gordon et al, 1975). In our study, procoagulant activities were neutralized by preincubation of cells with an anti-TF mAb, showing that TF contributes to the procoagulant status of lung cancer, and may therefore promote metastatic processes caused by products of the coagulation cascade. This contention is supported by our finding of higher levels of TF in cell lines derived from metastatic lesions or from malignant cells in pleural effusion. Although we did not determine the functional capacity of TF Ag recognized immunohistochemically in this study, Contrino et al (1994) examined TF expression in breast cancer using the same mAb as we used and reported that functional TF and antigenic TF were detected in parallel in various carcinomas. Therefore, we suggest that the TF Ag recognized immunohistochemically in this study is functionally active and that lung cancer cells positive for TF may have high coagulant activity, leading to high metastatic potential.

In conclusion, NSCLC cells produced various levels of TF. TF might modulate cancer cell behaviour and might be involved in the spread of lung cancer. Further studies of TF may provide new insights into the characteristics of lung cancer.

### ACKNOWLEDGEMENTS

The authors thank Dr Vernon L. Moore (Merck Research Laboratories, Rahway, NJ), Dr Takatoshi Koyama and Dr Azuma Watanabe (Tokyo Medical and Dental University, Tokyo, Japan) for their critical review of the manuscript. The skillful technical assistance of Ms Seiko Ohta is gratefully acknowledged.

### REFERENCES

Adamson AS, Francis JL, Witherow RO and Snell ME (1994*a*) Procoagulant properties of benign and malignant prostatic tissue. *Br J Urol* **74**: 204–209

Adamson AS, Luckert P, Pollard M, Snell ME, Amirkhosravi M and Francis JL (1994*b*) Procoagulant activity may be a marker of the malignant phenotype in experimental prostate cancer. *Br J Cancer* **69**: 286–290

Bromberg ME, Konigsberg WH, Madison JF and Pawashe A (1995) Tissue factor promotes melanoma metastasis by a pathway independent of blood coagulation. *Proc Natl Acad Sci USA* **92**: 8205–8209

Callander NS, Varki N and Rao LV (1992) Immunohistochemical identification of tissue factor in solid tumors. *Cancer* **70**: 1194–1201

Contrino J, Hair G, Kreutzer DL and Rickles FR (1996) In situ detection of tissue factor in vascular endothelial cells: correlation with the malignant phenotype of human breast disease. *Nature Med* **2**: 209–215

Contrino J, Hair GA, Schmeizl MA, Rickles FR and Kreutzer DL (1994) In situ characterization of antigenic and functional tissue factor expression in human tumors utilizing monoclonal antibodies and recombinant factor VIIa as probes. *Am J Pathol* **145**: 1315–1322

Drake TA, Morrissey JH and Edgington TS (1989) Selective cellular expression of tissue factor in human tissues. Implications for disorders of hemostasis and thrombosis. *Am J Pathol* **134**: 1087–1097

Esumi N, Fan D and Fidler IJ (1991) Inhibition of murine melanoma experimental metastasis by recombinant desulfatohirudin, a highly specific thrombin inhibitor. *Cancer Res* **51**: 4549–4556

Fair DS and MacDonald MJ (1987) Cooperative interaction between factor VII and cell surface-expressed tissue factor. *J Biol Chem* **262**: 11692–11698

Gasic GJ (1984) Role of plasma, platelets, and endothelial cells in tumor metastasis. *Cancer Metast Rev* **3**: 99–114

Gordon SG, Franks JJ and Lewis B (1975) Cancer procoagulant: a factor X-activating procoagulant from malignant tissue. *Thromb Res* **6**: 127–137

Gunji Y and Gorelik E (1988) Role of fibrin coagulation in protection of murine tumor cells from destruction by cytotoxic cells. *Cancer Res* **48**: 5216–5221

Hermanek P and Sobin LH (eds) (1992) UICC TNM *Classification of Malignant Tumours*, 4th edn, 2nd revision. Springer-Verlag: Berlin

Hiraki S, Miyai M, Seto T, Tamura T, Watanabe Y, Ozawa S, Ikeda H, Nakada Y, Oonoshi Y and Kimura I (1982) Establishment of human continuous cell lines from squamous cell, adeno and small cell carcinoma of the lung and the results of heterotransplantation. *Haigan* **22**: 53–57

Itoh H, Kataoka H, Koita H, Nabeshima K, Inoue T, Kangawa K and Koono M (1991) Establishment of a new human cancer cell line secreting protease nexin-II/amyloid beta protein precursor derived from squamous-cell carcinoma of lung. *Int J Cancer* **49**: 436–443

Kishimoto N (1992) Studies of cell biology and chemotherapy of lung cancer using tissue culture techniques. Part 2. Biological characteristics of five newly establishment small cell lung cancer cell lines. *Okayama Igakkai Zasshi* **104**: 905–913

Koyama T, Nishida K, Ohdama S, Sawada M, Murakami N, Hirosawa S, Kuriyama R, Matsuzawa K, Hasegawa R and Aoki N (1994) Determination of plasma tissue factor antigen and its clinical significance. *Br J Haematol* **87**: 343–347

Mackman N (1995) Regulation of the tissue factor gene. *FASEB J* **9**: 883–889

Morrissey JH, Fair DS and Edgington TS (1988) Monoclonal antibody analysis of purified and cell-associated tissue factor. *Thromb Res* **52**: 247–261

Mountain CF (1993) Lung cancer staging classification. *Clin Chest Med* **14**: 43–53

Mueller BM, Reisfeld RA, Edgington TS and Ruf W (1992) Expression of tissue factor by melanoma cells promotes efficient hematogenous metastasis. *Proc Natl Acad Sci USA* **89**: 11832–11836

Nemerson Y (1988) Tissue factor and hemostasis. *Blood* **71**: 1–8

Ornstein DL, Zacharski LR, Memoli VA, Kisiel W, Kudryk BJ, Hunt J, Rousseau SM and Stump DC (1991) Coexisting macrophage-associated fibrin formation and tumor cell urokinase in squamous cell and adenocarcinoma of the lung tissues. *Cancer* **68**: 1061–1067

Rehemtulla A, Pepe M and Edgington TS (1991) High level expression of recombinant human tissue factor in Chinese hamster ovary cells as a human thromboplastin. *Thromb Haemostas* **65**: 521–527

Ruf W and Edgington TS (1991) An anti-tissue factor monoclonal antibody which inhibits TF. VIIa complex is a potent anticoagulant in plasma. *Thromb Haemostas* **66**: 529–533

Ruf W, Rehemtulla A and Edgington TS (1991) Antibody mapping of tissue factor implicates two different exon-encoded regions in function. *Biochem J* **278**: 729–733

Smith BT (1977) Cell line A549: a model system for the study of alveolar type II cell function. *Am Rev Respir Dis* **115**: 285–293

Wilcox JN, Smith KM, Schwartz SM and Gordon D (1989) Localization of tissue factor in the normal vessel wall and in the atherosclerotic plaque. *Proc Natl Acad Sci USA* **86**: 2839–2843

Zhang Y, Deng Y, Luther T, Muller M, Ziegler R, Waldherr R, Stern DM and Nawroth PP (1994) Tissue factor controls the balance of angiogenic and antiangiogenic properties of tumor cells in mice. *J Clin Invest* **94**: 1320–1327

© Cancer Research Campaign 1999
